# Upregulation of microRNA-25 associates with prognosis in hepatocellular carcinoma

**DOI:** 10.1186/1746-1596-9-47

**Published:** 2014-03-04

**Authors:** Zhong-xue Su, Juan Zhao, Zhong-hou Rong, Wen-mao Geng, Ya-guang Wu, Cheng-kun Qin

**Affiliations:** 1 Department of Hepatobiliary Surgery, ShanDong Provincial Hospital affiliated to ShanDong University, 324 JingWu Road, Jinan, ShanDong Province 250021, China; 2 Department of Hematology, ShanDong Provincial Hospital affiliated to ShanDong University, Jinan, ShanDong Province 250021, China

## Abstract

**Background:**

Accumulating evidence has shown that up-regulation of microRNA-25(miR-25) is associated with the prognosis of several types of human malignant solid tumors. However, whether miR-25 expression has influence on the prognosis of hepatocellular carcinoma (HCC) is still unknown.

**Methods:**

The differentially expressed amount of the miR-25 was validated in triplicate by quantitative reverse-transcription polymerase chain reaction (qRT-PCR). Survival rate was analyzed by log-rank test, and survival curves were plotted according to Kaplan–Meier. Multivariate analysis of the prognostic factors was performed with Cox regression model.

**Results:**

The expression of miR-25 was significantly upregulated in HCC tissues when compared with adjacent normal tissues (p<0.0001). Patients who had high miR-25 expression had a shorter overall survival than patients who had low miR-25 expression (median overall survival, 31.0 months versus 42.9 months, p=0.0192). The multivariate Cox regression analysis indicated that miR-25 expression (HR=2.179; p=0.001), TNM stage (HR=1.782; p=0.014), and vein invasion (HR=1.624; p=0.020) were independent prognostic factors for overall survival.

**Conclusion:**

Our data suggests that the overexpression of miR-25 in HCC tissues is of predictive value on poor prognosis.

**Virtual slide:**

The virtual slide(s) for this article can be found here: http://www.diagnosticpathology.diagnomx.eu/vs/1989618421114309

## Introduction

Hepatocellular carcinoma (HCC) is one of the most malignant solid tumors and is the second leading cause of cancer-related mortality [1]. The established risk factors of HCC are viral hepatitis, alcohol abuse, and non-alcoholic fatty liver disease [2]. HCC is often diagnosed at an advanced stage, and it is not amenable to standard chemotherapy and is resistant to radiotherapy. In most cases, surgical resection and liver transplantation remain the only curative treatment options. Frequent tumor metastasis and recurrence after surgical intervention lead to the dismal outcome of patients with HCC. The 5-year survival rate of patients with HCC is approximately 5%, and over 650,000 people die of HCC each year worldwide [3]. The prediction of the prognosis and accurate patient stratification are crucial to optimize personalised treatment. This is currently performed by several staging scores, including the Barcelona Clinic Liver Cancer (BCLC) stage and the Cancer of the Liver Italian Program (CLIP) score [4,5]. Modifications of these staging systems by the addition of new biomarkers, in particular those better reflecting tumor aggressiveness, are likely to improve the prognostic assessment of HCC patients and could therefore fulfill a clinical need. Researchers have found some new prognostic markers of HCC, such as squamous cellular carcinoma antigen, SOX9, and L1 cell adhesion molecule [6-9].

The microRNAs (miRNAs) are a class of highly conserved short noncoding RNAs, which suppress protein expression by inhibiting translation or inducing mRNA degradation by binding to the 3′UTR of target mRNAs[10,11]. Beyond the involvement in diverse biological processes, it has been well demonstrated that deregulation or dysfunction of miRNAs can contribute to cancer development [12,13].

MiR-25 is a member of the miR-106b~25 cluster, which includes miR-106b, miR-93 and miR-25, that is located within intron 13 of the minichromosome maintenance protein 7(MCM7) gene on chromosome 7q22.1 [14,15]. Accumulating evidence has shown that up-regulation of miR-25 is associated with the prognosis of several human malignant solid tumors, including those of the stomach, ovary and prostate [16-18].

Recently, Li Y et al. found that the miR-106b~25 cluster was overexpressed in HCC tissues as well as cell lines, suggesting an important role of miR-106b~25 cluster in carcinogenesis and development of HCC [19]. However, the clinical relevance of miR-25 has not been studied yet, and whether miR-25 expression has influence on the prognosis of HCC is still unknown. Therefore, in the present study, we investigated the feasibility of miR-25 as a novel prognostic biomarker for HCC.

## Materials and methods

### Patients and Tissue Specimens

A total of 131 fresh specimens of HCC and paracarcinomatous liver tissues were obtained from 131 patients who underwent hepatic resection at Provincial Hospital affiliated to ShanDong University between June 2006 and July 2012. Among all the subjects, the mean follow-up period was 47 months (from 11 months to 65 months), and there was no censored case. All these tissues were immediately stored in liquid nitrogen and kept freshly frozen at − 80°C. Written informed consent was accomplished from the patients for publication of this study and any accompanying images. Study protocol was approved by the Ethics Committee of Provincial Hospital affiliated to ShanDong University. Before surgical therapy, none of the patients had received neoadjuvant chemotherapy, radiation therapy, or immunotherapy. Related important clinical information, including age, gender, serum level of α-fetoprotein (AFP), liver cirrhosis, Hepatitis B virus infection, tumor diameter, number of tumor nodules, and TNM stage were collected from each patient’s medical records. Clinical staging was performed according to the 2002 American Joint Committee on Cancer/International Union Against Cancer TNM staging system [20]. Patient characteristics were shown in Table 1.

**Table 1:** Relationship between miR-25 expression level and clinicopathologic parameters of hepatocellular carcinoma

**Factor**	**No.**	**miR-25 expression**	**P value**
Gender			
Male	81	8.34 ± 4.85	
Female	50	8.11 ± 4.63	0.62
Age at diagnosis(year)			
≤50	53	8.01 ± 5.11	
>50	78	8.49 ± 3.89	0.21
Tumor size(cm)			
≤5	62	7.99 ± 5.13	
>5	69	9.01 ± 3.79	0.13
Tumor number			
Solitary	70	8.13 ± 4.34	
Multiple	61	8.57 ± 4.89	0.51
TNM stage			
I-II	72	6.34 ± 3.12	
III-IV	59	11.01 ± 5.61	0.01
Vein invasion			
Presence	79	9.12 ± 4.89	
Absence	52	7.31 ± 4.19	0.24
Cirrhosis			
Negative	44	7.12 ± 3.19	
Positive	87	8.89 ± 5.38	0.17
Hepatitis B virus infection			
Negative	42	6.17 ± 4.65	
Positive	89	9.21 ± 4.13	0.09
Serum AFP level (ng/ml)			
≤25	54	6.02 ± 3.87	
>25	77	10.99 ± 5.44	0.02

AFP,α-fetoprotein.

### RNA isolation and quantitative RT-PCR of miRNA-25

Total RNA was isolated from frozen specimen by homogenizing tissue in Trizol reagent (Invitrogen, Carlsbad, CA, USA), according to the manufacturer’s instructions. The purity and concentration of RNA were determined using NanoDrop 1000 spectrophotometer (Thermo Scientific, Wilmington, DE, USA). The differentially expressed amount of the miR-25 was validated in triplicate by quantitative reverse-transcription polymerase chain reaction (qRT-PCR). Briefly, 2 ug of RNA was added to RT reaction, and then, the cDNA served as the template for amplification of PCR with sequence-specific primers (Sangon Biotech, Shanghai, China) using SYBR PrimeScript miRNA RT-PCR kit (Takara Biotechnology Co. Ltd, Dalian, China) on the 7500 Real-Time PCR systems (Applied Biosystems, Carlsbad, CA, USA). The PCR cycling profile was denatured at 95°C for 30 s, followed by 40 cycles of annealing at 95°C for 5 s, and extension at 60°C for 34 s. Small nucleolar RNA U6 was used as an internal standard for normalization. The cycle threshold (C_T_) value was calculated. The 2^-ΔCT^ (ΔC_T_=C_TmiR25_-C_TU6 RNA_) method was used to quantify relative amount of miR-25.

### Statistical analysis

Statistical analyses were performed using SPSS 13.0 soft-ware (Chicago, Ill., USA) and GraphPad Prism 5 (GraphPad Software Inc., CA, USA). The comparison of the expression level of miR-25 between HCC tissues and adjacent normal tissues was performed using the two-sample Student’s t test. The correlation between the expression level of miR-25 and clinicopathological characters was assessed with the two-sample Student’s t test. Survival rate was analyzed by log-rank test, and survival curves were plotted according to Kaplan–Meier. Multivariate analysis of the prognostic factors was performed with Cox regression model. All tests were two tailed and results with P<0.05 were considered statistically significant.

## Results

### The expression level of miR-25 in HCC samples and its relationship with clinicopathological characteristics

**Figure 1:**
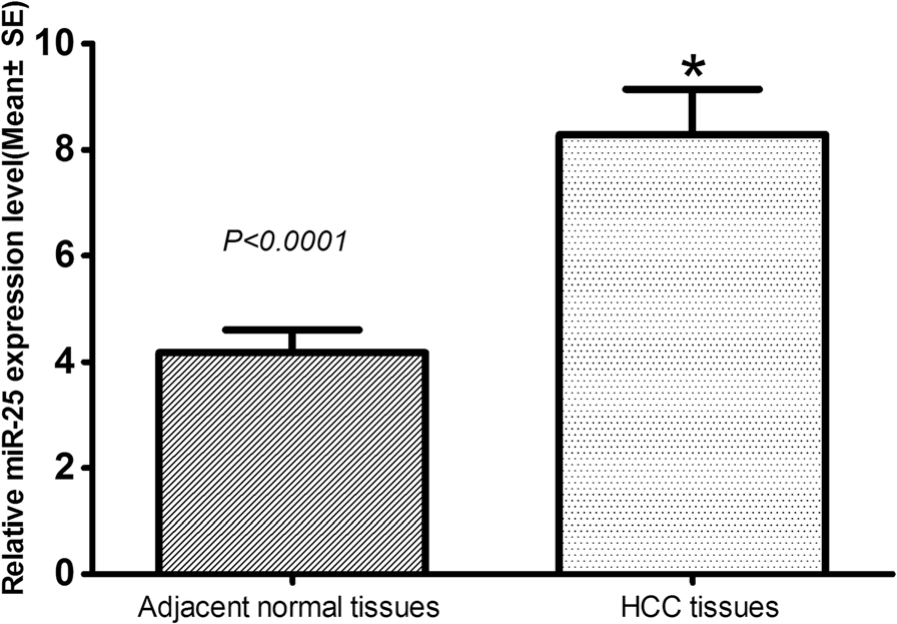
**Comparison of miR-25 expression levels between hepatocellular carcinoma tissues and adjacent normal tissues.** Analysis using the two sample Student’ s t test showed that the relative expression levels of miR-25 in the hepatocellular carcinoma tissues were significantly higher than those in adjacent normal tissues (p<0.0001) SE, Standard Error.

The expression of miR-25 was significantly upregulated in HCC tissues (mean relative expression level: 8.287, SD=4.711) when compared with adjacent normal tissues(mean relative expression level: 4.177, SD=2.386, P<0.0001, Figure 1). The relationship between miR-25 expression level with clinicopathological variables of HCC patients was shown in Table 1. The relative miR-25 expression levels were significantly positively correlated with serum AFP (p=0.02) and TNM stage (p=0.01).

However, there was no significantly correlation of miR-25 expression with other clinical features such as gender (p=0.62), age (p=0.21), liver cirrhosis (p=0.17), Hepatitis B virus infection (p=0.09), vein invasion (p=0.24), tumor diameter (p=0.13), or number of tumor nodules (p=0.51).

### The expression levels of miR-25 correlate with prognosis of patients with HCC

**Figure 2:**
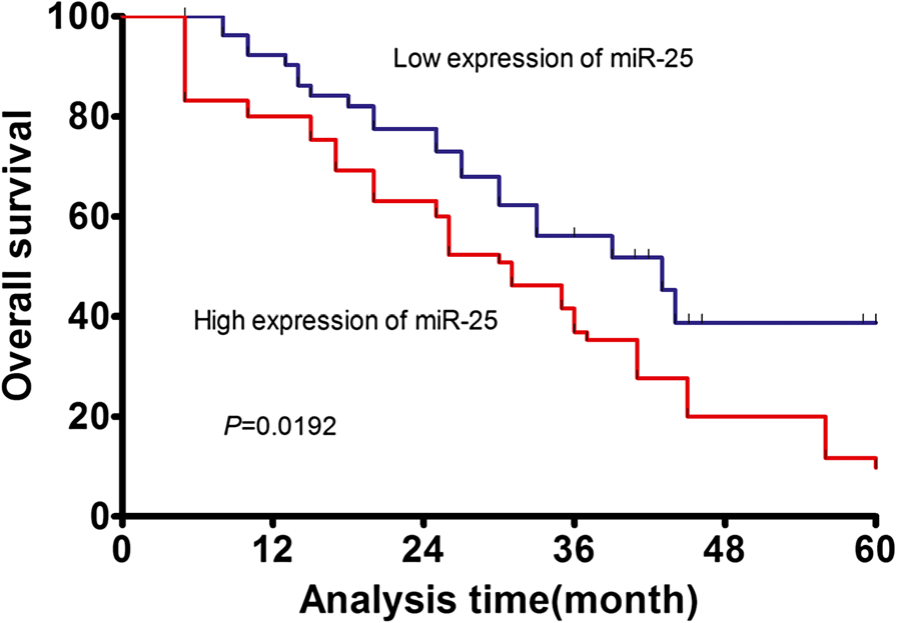
**Survival analysis of 131 hepatocellular carcinoma patients by Kaplan-Meier method.** Overall survival rate in patients with high miR-25 expression was significantly lower than that in patients with low miR-25 expression (p=0.0192).

Considering that the level of miR-25 expression was significantly correlated with TNM stage and serum AFP level, we hypothesized that miR-25 might affect the prognosis of HCC patients. To confirm this possibility, the miR-25 expression level and the prognosis of patients with HCC were analyzed by using the Kaplan–Meier method. The results indicated that patients who had high miR-25 expression had a shorter overall survival than patients who had low miR-25 expression (median overall survival, 31 months versus 42.9 months, p=0.0192; Figure 2). The multivariate Cox regression analysis indicated that miR-25 expression (HR=2.179; p=0.001), TNM stage (HR=1.782; p=0.014), and vein invasion (HR=1.624; p=0.020) were independent prognostic factors for overall survival (shown in Table 2).

**Table 2:** multivariate analyses of prognostic factors in hepatocellular carcinoma

**Variable**	**Subset**	**Overall survival**		
		**HR**	**95% CI**	**P**
miR-25 expression	High/low	2.179	1.876-4.335	0.001
Gender	Male/female	0.982	0.901-1.003	0.652
Age at diagnosis	>50/≤50	1.231	0.922-1.523	0.212
Tumor size	>5/≤5	1.479	0.991-2.127	0.090
Tumor number	Multiple/solitary	1.854	0.823-2.132	0.121
TNM stage	III-IV/I-II	1.782	1.389-2.993	0.014
Vein invasion	Presence/absence	1.624	1.233-1.981	0.020
Cirrhosis	Positive/negative	1.027	0.876-1.535	0.320
Hepatitis B virus infection	Positive/negative	1.002	0.892-1.337	0.417
Serum AFP level	>25/≤25	1.377	0.882-1.335	0.398

CI, confidence interval; HR, hazard ratio; miR-25, microRNA-25.

## Discussion

Although a growing number of novel treatment strategies have been developed for HCC, such as molecular targeted therapy and gene therapy, to our disappointment, satisfactory therapeutic outcomes have not been achieved [21,22]. Considering that the survival rate of HCC is still low, further identification of new prognostic markers remains important for the prevention and treatment of HCC.

The discovery that noncoding components of the genome, including microRNA, can contribute to the pathogenesis of cancer has led investigators to contemplate using these molecules to guide clinical decision making [23]. So far, there are more than 1000 microRNAs annotated by the latest version of miRBase. The expression of miRNAs is remarkably deregulated in HCC, strongly suggesting that miRNAs are involved in the initiation and progression of this disease. MiR-25 is a member of the miR-106b~25 cluster, which includes miR-106b, miR-93 and miR-25, that is located within intron 13 of the minichromosome maintenance protein 7 (MCM7) gene on chromosome 7q22.1 [14]. Previous studies have shown that the expression of miR-25 was up-regulated significantly in human stomach cancer, ovarian cancer, and prostate cancer [16-18]. In the study by Kim BH et al., miR-25 was found to be up-regulated in human gastric carcinoma tissues when compared to adjacent non-neoplastic tissues. The high expression of miR-25 in gastric carcinoma tissues may be a high risk factor associated with tumor penetration through serosa, lymph node metastasis, distant metastasis, and poor long-term survival in patients undergoing radical resection and adjuvant systemic chemotherapy [16]. Poliseno L et al. found that miR-106b~25 cluster was aberrantly overexpressed in human prostate cancer, which potentiated cellular transformation both in vitro and in vivo. They demonstrated that the intronic miR-106b~25cluster cooperated with its host gene MCM7 in cellular transformation both in vitro and in vivo [17]. Zhang H et al. have found that miR-25 was strongly up-regulated in ovarian cancer tissue versus adjacent non-tumor tissue. The expression levels of miR-25 in ovarian cancer cell lines were similar with ovarian cancer samples compared with the normal ovarian epithelial cells. Overexpression of miR-25 in ovarian cancer cells enhanced cell proliferation whereas down-regulation of miR-25 induced apoptosis. The effects of miR-25 abrogation were partly mediated by the intrinsic apoptosis pathway. Many pro-apoptotic proteins such as Bim, Bax and caspase-3 were up-regulated after transfection [18]. However, investigators also found that expression of miR-25 was down-regulated in other cancer. Li Q et al. found that the expression of miR-25 was down-regulated in colon cancer, and miR-25 might suppress the proliferation and migration of colon cancer cells as a tumor suppressor gene in vitro and in vivo [24]. Therefore, we speculate that the function of miR-25 is tissue specific.

Li Y et al. have found that miR-25 was strongly up-regulated in HCC tissue when compared with the corresponding paired non-tumor samples. However, the clinical significance of miR-25 gene expression in HCC remains unclear. In the present study, we found that miR-25 expression was proven to be associated with advanced TNM stage, suggesting that miR-25 might be involved in the carcinogenesis and metastasis of HCC. More importantly, we proved that patients with a high expression of miR-25 tended to have shorter survival than patients with lower levels, indicating that high miR-25 level is a marker of poor prognosis for patients with HCC. However, the precise molecular mechanisms behind the altered expression of miR-25 in HCC and its function are not very clear. In the study by Li Y et al., knock-down studies for the miR-106b-25cluster, which includes miR-106b, miR-93 and miR-25, showed that the expression of the cluster was necessary for cell proliferation and for anchorage independent growth [19]. Additional studies are needed to more clearly and comprehensively articulate the molecular mechanisms of both the cause and the effects of altered expression of miR-25 in the development and/or progression of HCC.

In summary, to the best of our knowledge, the present study is the first to report the differential expression of miR-25 in HCC and the possible use of miR-25 as a novel prognostic marker in HCC. The present findings demonstrate high expression of miR-25 in HCC tissue, which is associated with a poor prognosis in HCC patients. Further studies are needed to elucidate the mechanisms of action of miR-25 in HCC.

## Competing interests

The authors declare that they have no competing interests.

## Authors’ contributions

ZXS and JZ carried out the experimental studies and drafted the manuscript. ZHR, WMG, and YGW carried out part of the experimental studies. CKQ designed the experiments and modified the manuscript. All authors read and approved the final manuscript.
